# Feature engineering with clinical expert knowledge: A case study assessment of machine learning model complexity and performance

**DOI:** 10.1371/journal.pone.0231300

**Published:** 2020-04-23

**Authors:** Kenneth D. Roe, Vibhu Jawa, Xiaohan Zhang, Christopher G. Chute, Jeremy A. Epstein, Jordan Matelsky, Ilya Shpitser, Casey Overby Taylor

**Affiliations:** 1 Johns Hopkins Malone Center for Engineering in Healthcare, Johns Hopkins University, Baltimore, MD, United States of America; 2 The Institute of Clinical and Translational Research, Johns Hopkins University, Baltimore, MD, United States of America; 3 Department of Computer Science, Johns Hopkins University Whiting School of Engineering, Baltimore, MD, United States of America; 4 Division of Health Sciences Informatics, Johns Hopkins University School of Medicine, Baltimore, MD, United States of America; 5 Division of General Internal Medicine, Johns Hopkins University School of Medicine, Baltimore, MD, United States of America; 6 Johns Hopkins University Applied Physics Laboratory, Laurel, MD, United States of America; 7 Department of Biomedical Engineering, Johns Hopkins University, Baltimore, MD, United States of America; George Mason University, UNITED STATES

## Abstract

Incorporating expert knowledge at the time machine learning models are trained holds promise for producing models that are easier to interpret. The main objectives of this study were to use a feature engineering approach to incorporate clinical expert knowledge prior to applying machine learning techniques, and to assess the impact of the approach on model complexity and performance. Four machine learning models were trained to predict mortality with a severe asthma case study. Experiments to select fewer input features based on a discriminative score showed low to moderate precision for discovering clinically meaningful triplets, indicating that discriminative score alone cannot replace clinical input. When compared to baseline machine learning models, we found a decrease in model complexity with use of fewer features informed by discriminative score and filtering of laboratory features with clinical input. We also found a small difference in performance for the mortality prediction task when comparing baseline ML models to models that used filtered features. Encoding demographic and triplet information in ML models with filtered features appeared to show performance improvements from the baseline. These findings indicated that the use of filtered features may reduce model complexity, and with little impact on performance.

## Introduction

Improved access to large longitudinal electronic health record (EHR) datasets through secure open data platforms [1] and the use of high-performance infrastructure [2] are enabling applications of sophisticated machine learning (ML) models in decision support systems for major health care practice areas. Areas with recent successes include early detection and diagnosis [3, 4] treatment [5, 6] and outcome prediction and prognosis evaluation [7, 8]. Relying on ML models trained on large EHR datasets, however, may lead to implementing decision-support systems as black boxes—systems that hide their internal logic to the user [9]. A recent survey of methods for explaining black box models highlights two main inherent risks [10]: (1) using decision support systems that we do not understand, thus impacting health care provider and institution liability; and (2) a risk of inadvertently making wrong decisions, learned from spurious correlations in the training data. This work takes a feature engineering approach that incorporates clinical expert knowledge in order to bias the ML algorithms away from the spurious correlations and towards meaningful relationships.

## Background

### Severe asthma as a case study

We explored severe asthma as a case study given the multiple limitations of current computational methods to optimize asthma care management. Documented limitations include: the low prediction accuracy of existing approaches to project outcomes for asthma patients, limitations with communicating the reasons why patients are at high risk, difficulty explaining the rules and logic inside an approach and a lack of causal inference capability to provide clear guidance on what patients could safely be moved off care management [11]. Incorporating clinical expert knowledge at the time that computational models are trained may help to overcome these limitations.

### Expert clinical knowledge and model performance

Incorporate expert knowledge into the computational model building process has potential to produce ML models that show performance improvements. One previous study, for example, found that including known risk factors of heart failure (HF) as features during training yielded the greatest improvement in the performance of models to predict HF onset [12]. Different from that approach, we use a feature engineering approach to incorporate clinical expert knowledge.

Our feature engineering approach involved first extracting triplets from a longitudinal clinical data set, ranking those triplets according to a discriminative score, and then filtering those triplets with input from clinical experts. Triplets explored in this work were laboratory results and their relationship to clinical events such as medical prescriptions (i.e., lab-event-lab triples).

The goal of this research was to apply the feature engineering approach with a severe asthma case study and to assess model performance for a range of ML approaches: gradient boosting [13], neural network [14], logistic regression and k-nearest neighbor. Non-zero coefficients were assessed as a metric of model complexity for two ML approaches: logistic regression and gradient boosting.

For each ML model, we conducted several experiments to understand the impact of ranking features based upon discriminative score and of filtering features with clinical input on model complexity and performance. To assess performance, we used measures of model accuracy and fidelity. Experiments were completed with a case study of patients with severe asthma in the MIMIC-III [1] dataset for a mortality prediction task.

## Methods

### Discovering triplets from longitudinal clinical data

First, we discovered triplets, defined as a lab-event-lab sequence where the value of a laboratory result is captured before and after a clinical event. These triplets occur within the context of an ICU stay. Clinical events captured in this study were medication prescriptions and clinical procedures. The ranking step used an information theoretic approach to calculate and associate a discriminative score for triplets. The filtering step involved input from clinical experts who filtered out triplets that were not considered relevant to asthma. The final list of ranked and filtered laboratory results were used to select or weight features in a range of machine learning models.

In order to discover triplets, laboratory results were pre-processed as follows:

1. Laboratory values were cleaned by merging laboratory result names according to the approach described in ref [17]. That work provided a file outlining bundled laboratory names (e.g., heart rate) that grouped name variations (e.g., pulse rate), abbreviations (e.g., HR) and, misspellings (e.g., heat rate) of the same concept [18]. In addition, there were circumstances where laboratory values consisted of both numerical and textual representations. In those cases, we converted the textual values to numbers according to simple rules (e.g., “≤1” converted “1”). Many laboratory result entries had values such as “error” which could not be converted. In those instances, entries were ignored.
2. Laboratory values were divided into a finite number of bins. Bin boundaries were defined by a clinical expert familiar with the normal ranges of each laboratory test. For tests where normal ranges were unknown, six dividers were defined based upon mean and standard deviation (i.e., *μ* − 2*σ*, *μ* − *σ*, *μ* − *σ*/2, *μ* + *σ*/2, *μ* + *σ*, *μ* + 2*σ*).

Next, triplets were discovered according to the following steps:

1. Laboratory value bins before and after a clinical event (i.e., a lab-event-lab triplet) were captured. A laboratory result could involve different clinical events—resulting in multiple triplets. In addition, each patient in our dataset could have multiple triplets. The amount of time between the clinical event and lab measurement also varies depending upon the lab. For each event, lab test time duration before and after each event were calculated. For each lab test, time duration before an event was defined as the time immediately after the prior lab test until (and including) the time of the event. The duration after an event was defined as the time immediately after the event until the next lab measure occurred. The start time for the first recorded lab measure for an individual, was defined as the start time for the ICU stay. Similarly, the end time for the last recorded lab measure, was the defined as the end time for the ICU stay.
2. Lab-event-lab triplets were categorized as no change, decreasing or increasing by assessing the laboratory value bin before and after the anchoring clinical event.
3. Cross tabulations where then performed for each triplet category (no change, decreasing, increasing) for two patient sub-groups (patients who died and patient that did not die).
4. Triplets with cross tabulation values of 10 or fewer were excluded from further analysis. We did this because small counts cannot reliability determine whether there is a statistically meaningful relationship.

### Ranking and filtering laboratory result features

We used an information theoretic approach to calculate discriminative scores for triplets and used those scores to rank and filter laboratory result features. In particular, we calculated mutual information [19] score, *MI*_*score*_ (Eq 1) to rank triplets that may be estimators of mortality. *MI*_*score*_ was chosen as simple and fast measure that can be used to highlight triplets that may be relevant for clinical experts. The *MI*_*score*_ is a measure of the marginal association of each triplet category with patient mortality sub-group, thus it is based on a table with three columns (no change, increase, decrease) and two rows (died or survived). An illustration of *MI*_*score*_ calculations for triplets is shown in (Table 1).

$$\begin{array}{c} MI_{score}=\sum P\left(XY\right)log\left(\frac{P(XY)}{P(X)P(Y)}\right) \end{array}$$ (1)

**Table 1:** A hypothetical case showing mutual information (*MI*_*score*_) calculation.

Drug B	No change (Lab A)	Increased (Lab A)	Decreased (Lab A)
Survived	12	11	12
Died	22	22	21
*MI*_*score*_ = 0.00034354			
Drug C	No change (Lab A)	Increased (Lab A)	Decreased (Lab A)
Survived	25	8	5
Died	12	23	27
*MI*_*score*_ = 0.11523			

Due to table dimensions (two by three), the *MI*_*score*_ will always be between 0 and log2 ≈ 0.6931. For this reason, we did not use normalized mutual information. While more sophisticated measures of association between features and outcomes, conditional on other features, such as conditional mutual information are potentially informative, they are also very challenging to evaluate in our setting. Given that our models include high dimensional feature sets, we choose this more simple measure.

After *MI*_*score*_’s were calculated for triplets, clinical experts hand-picked the subset that were clinically relevant to asthma. The selected triplets were then used to *filter* laboratory result features. Filtered features were those laboratory tests that were represented among the clinically meaningful triplets. The filtered laboratory result features were used in experiments described in the “Evaluation” section.

We also calculated a composite discriminative score that was used to *rank* laboratory results features. For each laboratory result represented among all triplets, a discriminative score was calculated by taking the sum of *MI*_*score*_’s from each triplet in which it appeared. The discriminative scores were used to rank laboratory result features used in experiments described in the “Evaluation” section.

### Machine learning models for longitudinal clinical datasets

For all machine learning models explored in this study (gradient boosting, neural network, logistic regression and k-nearest neighbors), time series data was used. KNN allows us to specify feature importance. The other models do not support the input of feature weights. However, we performed experiments selecting subsets of the most important features.

For all four models, we normalized the training data and normalized features by removing the mean and scaling to unit variance. Normalization is done to prevent biasing the algorithms. For example, many algorithms tend to sum together features, which would cause bias towards features with a wide range of values.

This same pipeline was used on the test dataset. 10-fold cross validations with a limited hyper parameter search was used to predict mortality. Baseline ML models and clinical input-informed ML models were created. Baseline ML models included 42 top ranked features according to discriminative score. Different from baseline ML models, clinical input-informed ML models included filtered features (i.e., a subset of laboratory result features determined to be clinically relevant). We used both feature selection and weighting approaches, depending on the modeling approach. For logistic regression, gradient boosting, and neural network (using shallow decision trees as week learners), we used feature weights to perform feature selection. For KNN, we used weighted distance as described by others [20] to do prediction.

## Evaluation

We evaluated model complexity and model performance with two characteristics of the feature engineering approach: ranking features by discriminative score, and filtering features according to input on which triplets are clinically meaningful. We used one measure of model complexity and two measures of performance (model accuracy and model fidelity). Our specific research questions were:

- What is the impact of our approach to rank laboratory result features according to discriminative score on model complexity? and on model performance (model accuracy and model fidelity)?
- What is the impact of our approach to filter features on model complexity? and on model performance (model accuracy and model fidelity)?
- What is the impact of encoding triplet information directly into the model on model complexity? and on model performance (model accuracy and model fidelity)?
- What is the model complexity and performance trade off of ranking laboratory result features according to discriminative score? of filtering features? and of encoding triplet information directly into the model?

**Fig 1:**
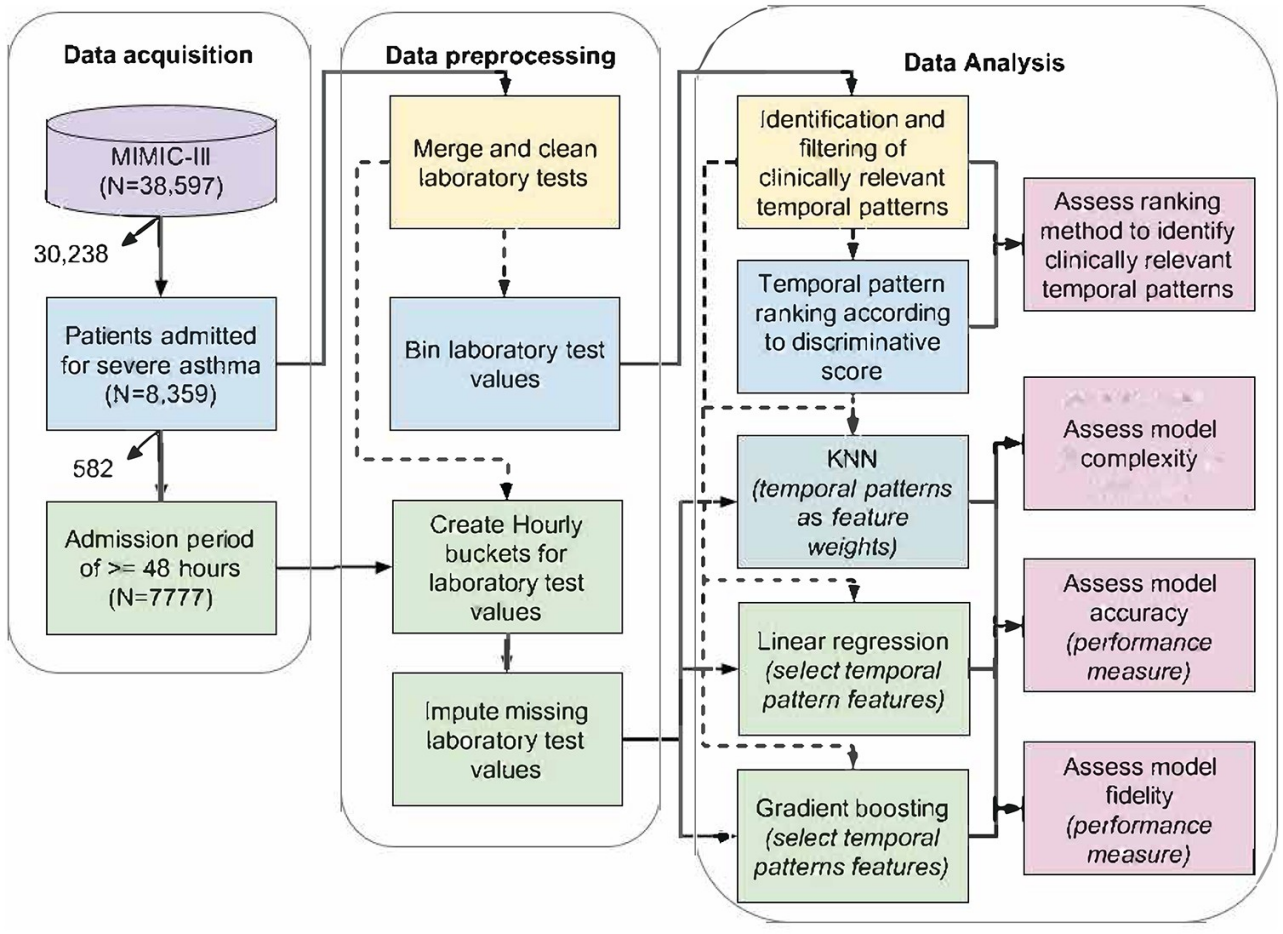
Summary of data acquisition, data pre-processing, and data analysis steps.

A summary of data acquisition and data pre-processing steps to enable these analyses, as well a description of what was analyzed is summarized in Fig 1 and below.

### Data source, study population and machine learning models

Previous work indicates that admission to the intensive care unit for asthma is a marker for severe disease [21]. Thus we chose to use the MIMIC-III (‘Medical Information Mart for Intensive Care’) public dataset [1] that includes de-identified health care data collected from patients admitted to critical care units at Beth Israel Deaconess Medical Center from 2001 to 2012.

Patients included in our analyses had an asthma diagnosis or medication to treat asthma according to criteria proposed by the eMERGE (Electronic Medical Records and Genomics) consortium [22]. We also use the in-hospital mortality labels defined in MIMIC-III for our case study task to predict whether a patient dies in the hospital. This task was treated as a binary classification problem. For the task to predict patient mortality, we narrowed our cohort to include only those patients included in a MIMIC III benchmark dataset [17] and with an admission period of ≥48 hours. We selected logistic regression, gradient boosting, neural network and KNN ML models that were trained as implemented by the scikit-learn Python package [14].

We considered gradient boosting models to be “black-box” due to the lack of direct interpretation with the use of boosted classifiers with multiple trees. Neural network models are also considered “black-box” given that there are often many layers of neurons and it is difficult to relate connection weights to specific concepts. For logistic regression, the coefficients have an interpretation in terms of log odds. KNN also offers some interpretability because we know the closest data points to the current query point being used for making a decision. Note, however that these notions of what is considered black-box may be appropriate in different contexts.

### Data subsets and machine learning experiments

To conduct our study, we performed experiments with laboratory results and data subsets that incrementally added encoded information on patient demographics, on clinical events from triplets, and on laboratory results from triplets. These four data subsets were used to train the ML models. The first *Labs* data subset comes directly from the MIMIC III benchmark dataset. It contained, for each patient, the sequence of laboratory results collected during their first ICU admission. The values at each hour over a 48 hour period were included. For each one hour period, we could have zero values, one value or more than one value. For each of the *f* × 48 slots (where *f* is the number of labs or features used in the machine learning experiment), we computed mean, min, max, standard deviation and skew. This created a total of *f* × 48 × 5 inputs to the ML models. We also normalized our datasets so that the mean is zero and variance is one. This was done using sklearn’s StandardScalar [14].

The second *Labs+demo* data subset added demographic information: age group at the time of admission to the ICU, race/ethnicity and sex. Age groups included: *<2*, *2 − 17*, *18 − 34*, *35 − 49*, *50 − 69* and *70+* years old. Race and ethnicity’s included: *white*, *black*, *asian*, *hispanic*, *multi* and *other*. The *other* category was used when we could not determine the group based on the MIMIC III entry. For sex, groups included *male* and *female*. For each patient, these values were repeated for all time slots.

The third *Labs+demo+events* data subset added a column for each clinical event (ie., drug prescriptions and procedures) from triplets considered clinically relevant. Each column includes a zero or a one, with one indicating that the clinical event was recorded during the time slot being considered, and zero otherwise.

The fourth *Labs+demo+events+triples* data subset duplicates columns from the *Labs* data subset and for a given time slot, replaces laboratory values with a zero if all clinical event values are zero. Otherwise, the laboratory values were left as-is.

The *Labs+demo+events* and *Labs+demo+events+triples* data subsets allowed us to examine the extent to which model complexity and performance was impacted by encoding triplet information. See “Machine learning experiments and analyses of model complexity and performance trade-off” for details on experiments with these data subsets.

### Analysis of triplet ranking

The ranking of triplets according to discriminative score was assessed by three co-authors (XZ, CGC, JAE) who manually reviewed the clinical relevance of all features in our severe asthma case study. A two-step strategy was applied. The first step was to evaluate whether the medication/procedure was generally known to or could conceivably have an impact (directly or indirectly) on a laboratory result (e.g., we consider arterial blood gas and pulmonary function test results to have medical relevance in asthma patients). The second step was to evaluate whether the combination is considered relevant to asthma case study (e.g., ‘Gauge Ordering’ does not indicate specific clinical uses). This process allowed us to filter lab-event-lab triplets that were not relevant to our case study. For top ranked triplets according to *MI*_*score*_, we calculated *precision* in the top *k* ranked triplets, i.e. the fraction of the count of individual triplets selected by our experts within the top *k* ranked patterns, divided by *k*. This set was used in the ML predictors explored in this study. All of steps to analyze the model complexity and performance trade-off were computational.

### Machine learning experiments and analyses of model complexity and performance

We conducted several experiments to assess the model complexity and performance of ML models. We used one measure of model complexity and two measures of performance (model accuracy and model fidelity). Complexity, accuracy and fidelity are three characteristics used to describe ML algorithms that have been summarized by others [9, 23].

#### Measuring ML model complexity

To assess the impact of using ranked laboratory result features on model complexity, we conducted experiments analyzing the number of non-zero coefficients with use of fewer than the baseline 42 features (*k* = 32, 16, 8, 4, and 2). In order to assess the impact of using filtered features on model complexity, we conducted experiments comparing the number of non-zero coefficients in the baseline ML model and in the ML models based on filtered features. In order to assess the impact of encoding triplet information into the model on model complexity, we compared the number of non-zero coefficients in ML model subsets that included triplet information (i.e., *Labs+demo+events* and *Labs+demo+events+triples*) to the data subset that includes laboratory results and demographic information (i.e., *Labs+demo*). This assessment was conducted for logistic regression and gradient boosting. We did not assess non-zero coefficients for neural network or KNN because the number of non-zero coefficients is not related to the complexity for these models. For all comparisons, we assessed the degree of difference.

#### Measuring ML model accuracy

Model accuracy was assessed by examining the extent to which ranking laboratory result features according to discriminative score and filtering features in ML models can accurately predict mortality. For models with ranked features and with filtered features, we also examined the extent to which encoding demographic information and triplet information influences model accuracy. Model fidelity was assessed by observing the extent to which ML models with filtered features (with and without encoding triplet information directly) are able to imitate the baseline ML predictors.

In order to assess the model accuracy of ranking according to discriminative score, we performed feature selection experiments with logistic regression, gradient boosting, and neural network models. For these experiments, we selected the top *k* = 2, 4, 8, 16, 32, and 42 laboratory results ranked according to model weights for gradient boosting and neural network models, and according to the sum of *MI*_*score*_ for logistic regression models. For KNN we did not assess performance changes when considering ranking according to discriminative score. For each experiment, receiver operating characteristic (ROC) curves and area under the ROC curve (AUC) were reported. The range of AUC values was also reported for feature selection experiments.

In order to assess the model accuracy of baseline and clinical input-informed ML models, for all three models we performed experiments with data subsets. Baseline ML models included models with 42 features and its data subsets. Filtered features were laboratory results represented among triplets determined to be clinically relevant. For logistic regression, gradient boosting, and neural network models, the *Labs+demo* data subset enabled assessing the influence of encoding demographic information on model accuracy. The *Labs+demo+events* and *Labs+demo+events+triples* data subsets enabled assessing the influence of encoding triplet information on model accuracy. KNN experiments were conducted with the *Labs* data subset only, so we did not assess the influence of encoding triplet information. For each experiment, ROC curves and AUC were reported.

#### Measuring ML model fidelity

Fidelity was assessed by examining the extent to which the clinical input-informed ML features (with and without triplet information) are able to accurately imitate our baseline ML predictors. We conducted experiments that enabled comparing clinical input-informed ML model performance to baseline ML model performance. For logistic regression, gradient boosting, and neural network models, clinical input-informed ML models were compared to baseline ML models for three data subsets: *Labs*, *Labs+demo* and *Labs+demo+events*. For KNN, we used the *Lab* data subset and compared the performance of models with filtered features (with and without weights) to the baseline ML model. We report the difference in *AUC* for clinical input-informed ML models with and without encoding triplets (i.e., the *Labs+demo+events* and *Labs+demo+events+triples* data subsets) compared to the baseline for logistic regression, gradient boosting, and neural network ML models.

## Results

### Triplet identification and ranking

We discovered 218 prescription and 535 procedure triplets with more than 10 instances. These two lists of triplets were sorted by *MI*_*score*_ prior to manual clinical review. Upon clinical review, we found that 82 triplets (27 prescription and 55 procedure triplets, see S1 and S2 Tables) were meaningful for our case study. Precision at *k* in a top-*k* problem for prescription and procedure events according to *MI*_*score*_ are shown in Table 2. Triplets used in this calculation are summarized in S3 and S4 Tables. For prescription triplets, precision at *k* = 3, 5, 10, and 20 in a top-*k* ranked list ranged from 20% to 67% i.e., the percentage of the triplets that were relevant to the case study. For procedure triplets, precision at *k* = 3, 5, 10, and 20 in a top-*k* ranged from 0% to 20%.

**Table 2:** Precision for top *k* prescription and procedure triplets.

Triplet event	*Precision*_*top*3_	*Precision*_*top*5_	*Precision*_*top*10_	*Precision*_*top*20_
Prescription	67.0%	40.0%	30.0%	20.0%
Procedure	0.0%	0.0%	0.0%	20.0%

The triplets were used to enable more interpretable ML models through their use to select and weight features. Eleven laboratory tests were represented among the 82 clinically meaningful triplets discovered at this step (i.e., filtered laboratory results, see S5 Table). The “Performance result: ML model accuracy” section shows how feature selection using the 11 laboratory tests (i.e., filtered features) impacted the ML result accuracy).

### Machine learning results

The ML experiments are based on a subset of 7777 patient records from the MIMIC III database that are also included in a benchmark dataset used by others [17]. This dataset of 7777 patients was divided into 6222 training cases (death rate 0.489) and 1555 for testing cases (death rate 0.494). An overview of the final dataset, data pre-processing and data analysis steps are shown in Fig 1 and Table 3. The rankings of the top 42 ranked laboratory tests used in logistic regression and gradient boosting model experiments are shown in S6 and S7 Tables.

**Table 3:** Summary of dataset.

Data	Total
# Admissions in the MIMIC-III (v1.4) database	58 576
# Admissions with asthma medication	8359
# Admissions with visit duration > = 48 hours	7777
# Training set of patients for the model	6222
# Testing set of patients the model	1555

### ML model complexity results

The ML models for logistic regression and gradient boosting for *k* ≤ 42 laboratory result features are illustrated in Tables 4 and 5. For both, model complexity decreased with use of fewer features informed by discriminative score (Tables 4 and 5). For logistic regression models, there was a -1.8 fold change in non-zero coefficients between with 16 and 8 features, a -2.8 fold change between models with 4 and 2 features. For gradient boosting models, there was -1.7 fold change in non-zero coefficients between models with 32 and 16 features, a -1.7 fold change between models with 8 and 4 features, and a -2.5 fold change for models with 4 and 2 features. All other model fold changes were 0 to 0.5 and were interpreted as no difference.

The use of clinical input-informed (filtered) features decreased model complexity for logistic regression and gradient boosting ML models. For logistic regression and gradient boosting models, they both had a -1.7 fold change in non-zero coefficients between the models with filtered features for *Labs* data subsets and the baseline model. There was no difference between models with filtered features for *Labs+demo* and *Labs+demo+events* data subsets, and the baseline model (i.e., fold changes were 0 to 0.5).

When examining the influence of encoding demographic and triplet information for models with non-filtered features, we found that model complexity decreased. Among logistic regression models, there was a -1.7 fold change in non-zero coefficients between models that encode demographic information (*Labs+demo*) compared to the baseline model (*Labs*), and a -1.6 fold change between models that encode triplet information (*Labs+demo+events*) compared to the baseline model. Among gradient boosting models, there was a -1.9 fold change in non-zero coefficients between models that encode demographic information (*Labs+demo*) compared to the baseline model, and a -2.0 fold change between models that encode triplet information (*Labs+demo+events*) compared to the baseline model.

When examining the influence of encoding demographic and triplet information for models with filtered features, the model complexity increased for one model that encoded triplet information. There was a 2.3 fold change for the gradient boosting model with filtered features for the *Labs+demo+events+triples* data subset compared to the baseline model. There were no differences between other models encoding demographic and triplet information with filtered features and the baseline model (i.e., fold changes were 0 to 0.5 for *Labs+demo* and *Labs+demo+events* data subsets).

**Table 4:** Trained logistic regression model parameters.

Features	Data subsets	Number of coefficients	Number of non-zero coefficients
2 features	Labs	480	123
4 features	Labs	960	344
8 features	Labs	1920	418
16 features	Labs	3840	770
32 features	Labs	7680	894
42 features	Labs	10080	1058
	Labs+demo	10224	619
	Labs+demo+events	11568	684
	Labs+demo+events+triples	-	-
11 filtered features	Labs	2640	614
	Labs+demo	2784	448
	Labs+demo+events	4128	609
	Labs+demo+events+triples	47568	686

**Table 5:** Trained gradient boosting model parameters.

Features	Data subsets	Number of coefficients	Number of non-zero coefficients
2 features	Labs	480	148
4 features	Labs	960	365
8 features	Labs	1920	612
16 features	Labs	3840	452
32 features	Labs	7680	778
42 features	Labs	10080	732
	Labs+demo	10224	384
	Labs+demo+events	11568	374
	Labs+demo+events+triples	-	-
11 filtered features	Labs	2640	431
	Labs+demo	2784	330
	Labs+demo+events	4128	468
	Labs+demo+events+triples	95136	995

### Performance result: ML model accuracy and fidelity

Model accuracy and fidelity for logistic regression, gradient boosting, and neural network ML models (top *k* = 2, 4, 8, 16, 32, 42 features, and 11 filtered features) are summarized in Table 6. The top performing models yielded *AUC*’s of 0.73 for logistic regression, 0.75 for gradient boosting, and 0.68 for neural network models. For KNN the baseline model accuracy was *AUC*_42_ = 0.57.

**Table 6:** Logistic regression, gradient boosting, and neural network: *AUC* of top *k* features.

Features	Data subsets	Logistic regression	Gradientboosting	Neural networks
2 features	Labs	0.54	0.60	0.57
4 features	Labs	0.55	0.63	0.55
8 features	Labs	0.56	0.63	0.57
16 features	Labs	0.60	0.66	0.61
32 features	Labs	0.64	0.68	0.64
42 features	Labs	0.64	0.69	0.63
	Labs+demo	0.73	0.74	0.67
	Labs+demo+events	0.73	0.75	0.65
	Labs+demo+events+triples	-	-	-
11 filtered features	Labs	0.64	0.68	0.62
	Labs+demo	0.72	0.73	0.67
	Labs+demo+events	0.73	0.74	0.68
	Labs+demo+events+triples	0.72	0.74	0.65

*AUC* = area under the receiver operating characteristic curve

**Fig 2:**
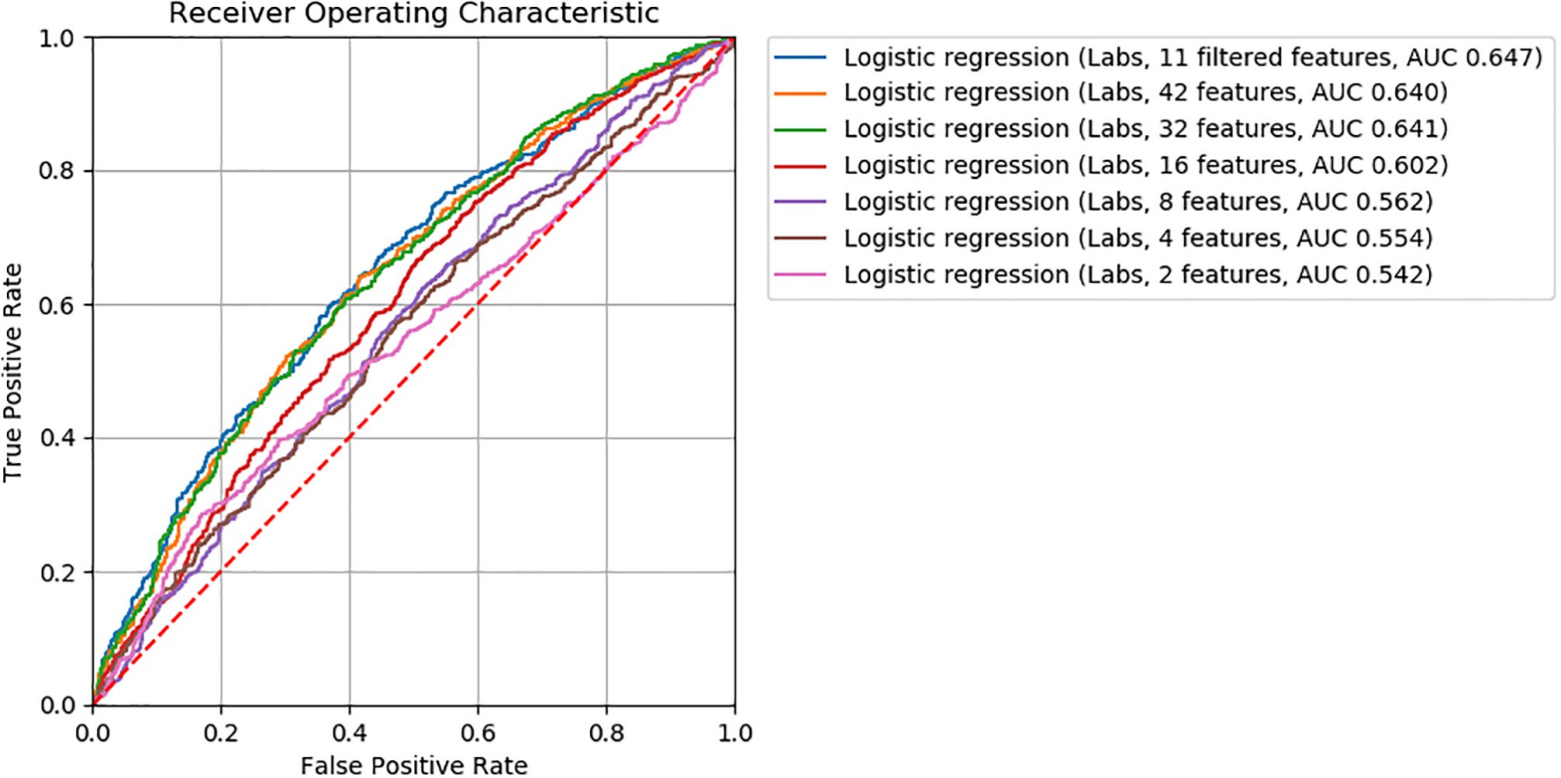
ROC curves for logistic regression classifiers.

**Fig 3:**
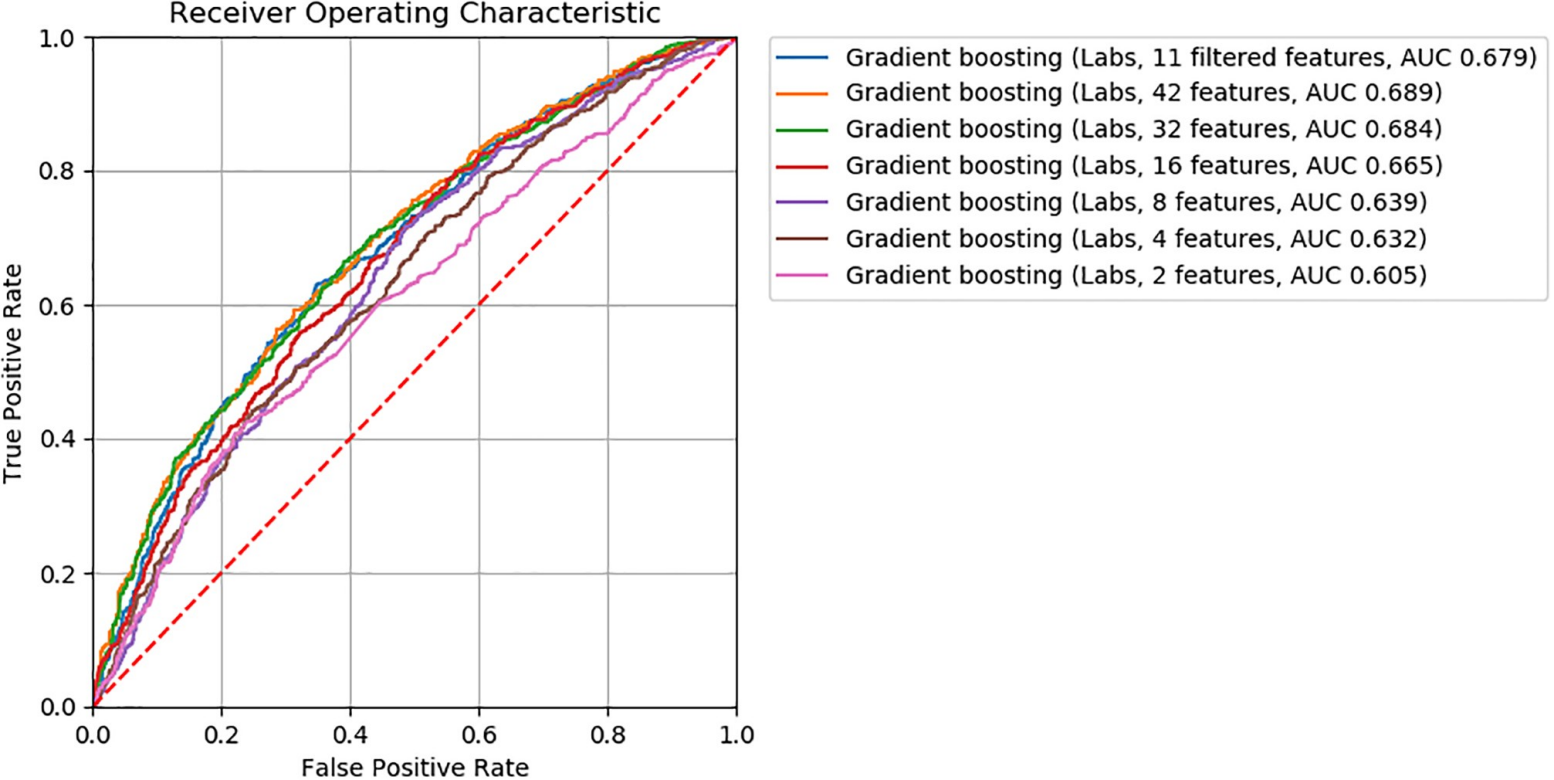
ROC curves for gradient boosting classifiers.

**Fig 4:**
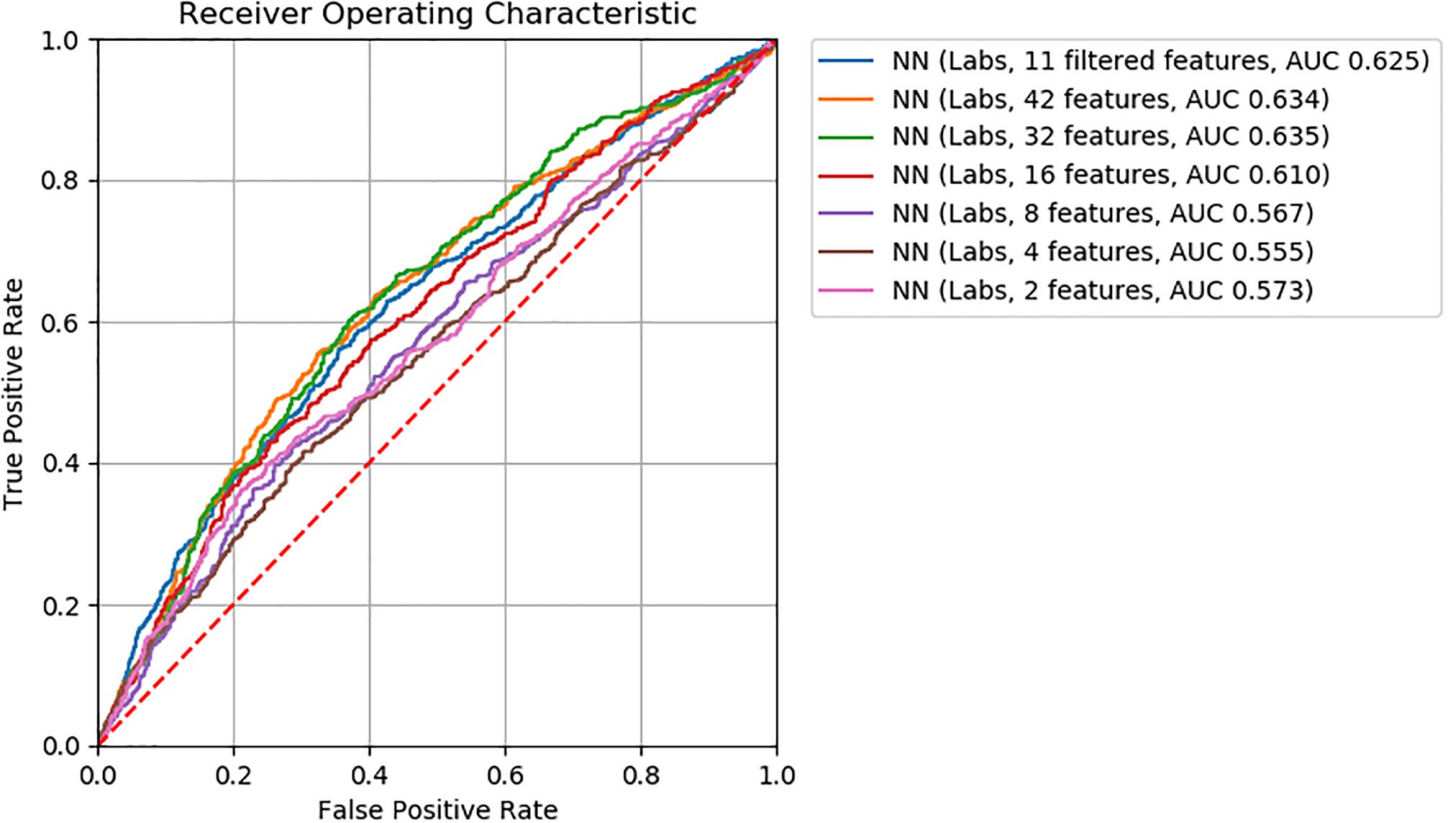
ROC curves for neural network classifiers.

For logistic regression, gradient boosting, and neural network ML models, we found model accuracy to be robust to feature removal informed by discriminative score (Figs 2, 3 and 4). We observed slightly lower *AUC*’s between models with 32 and 16 features (*ΔAUC*_32‖16_ = 0.04, 0.02, and 0.03 for logistic regression, gradient boosting, and neural network models, respectively). Step-wise feature removal otherwise yielded negligible differences (i.e., *ΔAUC* ≤ 0.01). Across all data subsets, the maximum difference from baseline was *ΔAUC*_*max*_ = 0.1 for logistic regression, *ΔAUC*_*max*_ = 0.09 for gradiant boosting, and *ΔAUC*_*max*_ = 0.06 for neural network models.

**Fig 5:**
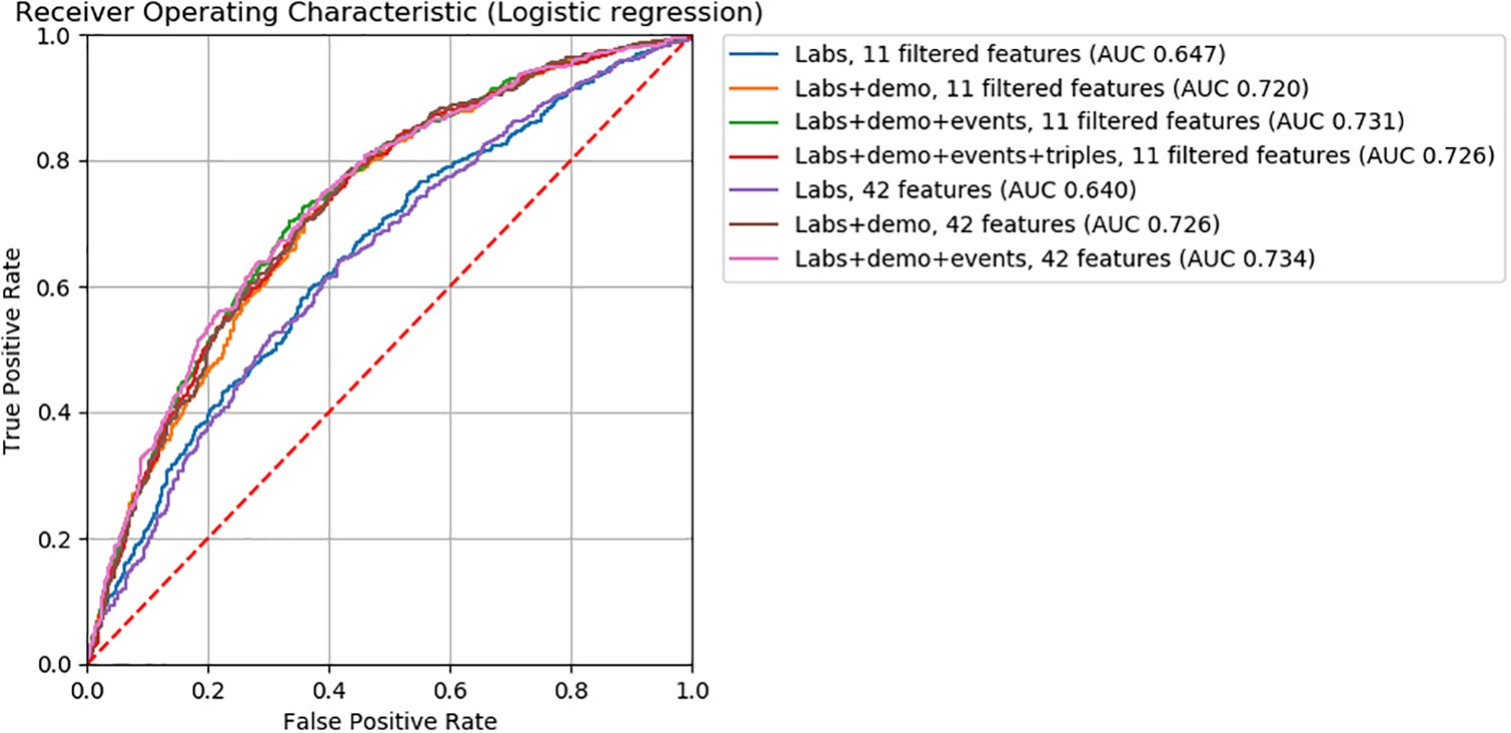
ROC curves for logistic regression classifiers.

**Fig 6:**
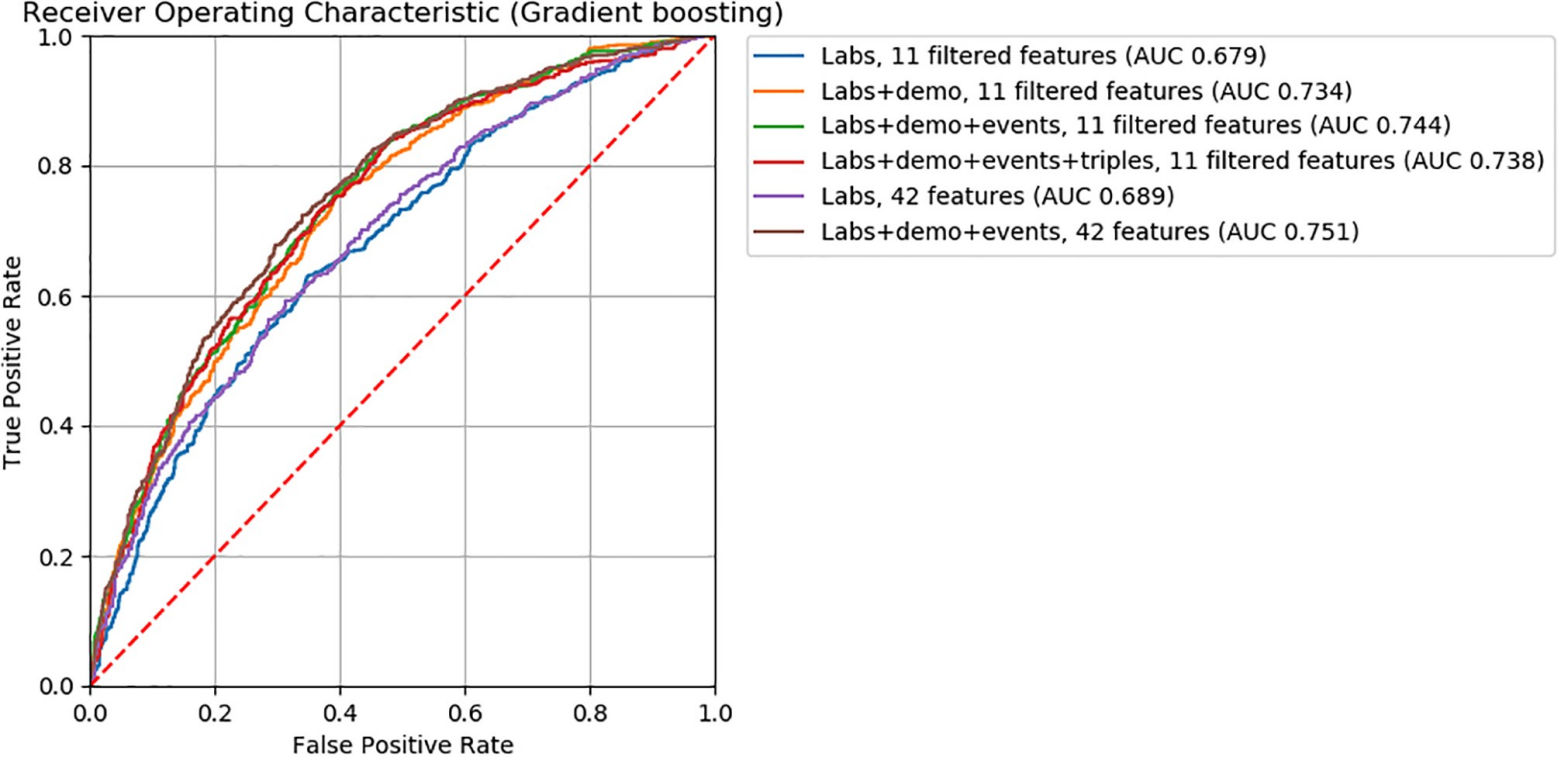
ROC curves for gradient boosting classifiers.

**Fig 7:**
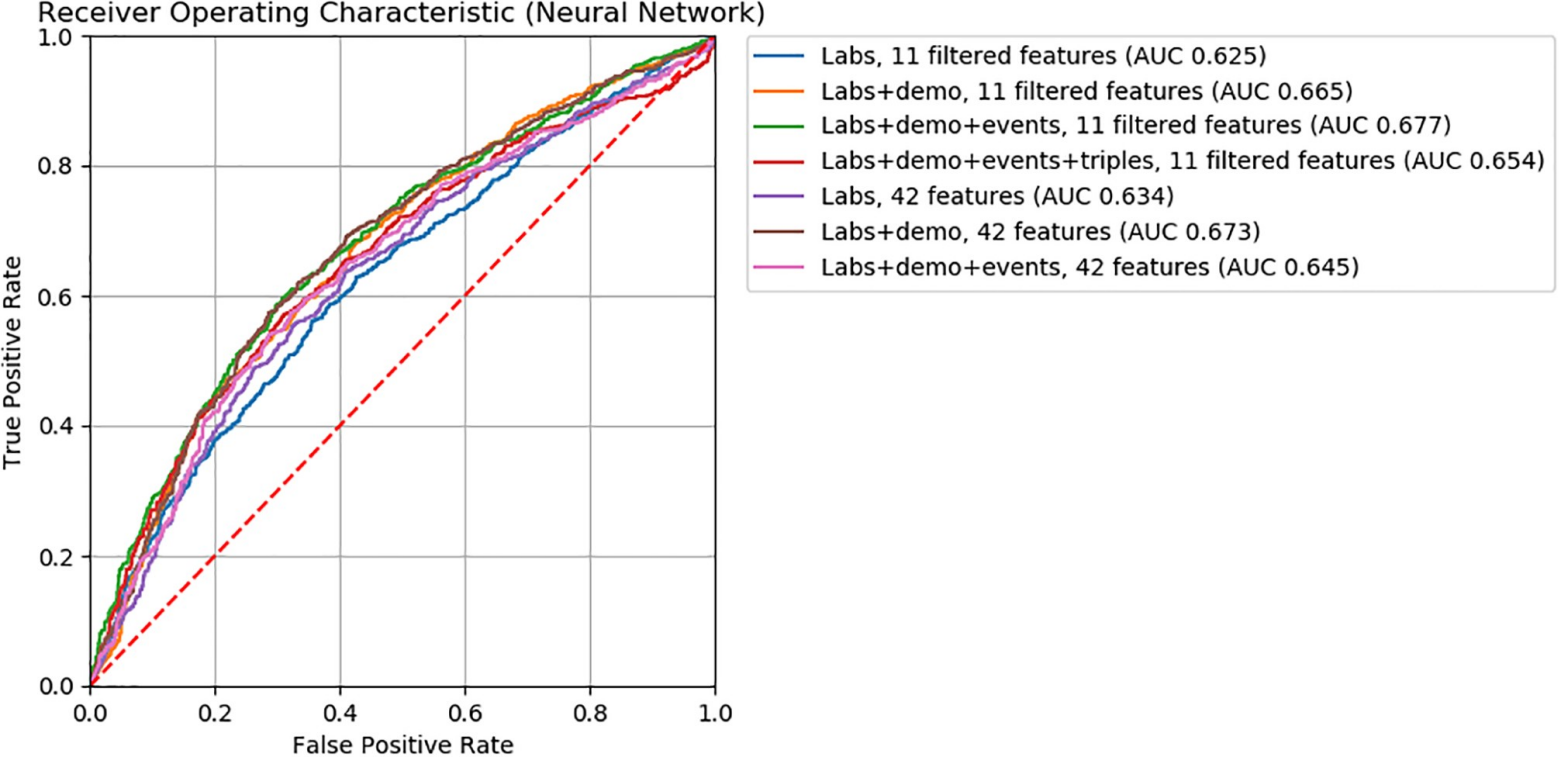
ROC curves for neural network classifiers.

For all ML models, the clinical input-informed ML features (i.e., filtered features) yielded comparable model accuracy to the baseline (Figs 5, 6 and 7). The top performing models with filtered features were 0.73 for logistic regression, 0.74 for gradient boosting, 0.68 for neural network, 0.54 for KNN_*unweighted*_, and 0.56 for KNN_*weighted*_ models. The magnitude of the difference in *AUC* from baseline models were >0.01 for the KNN_*unweighted*_ model that achieved lower but comparable accuracy (*ΔAUC* = 0.03), and for the neural network model for the Labs+demo+events data subset achieved higher but comparable accuracy (*ΔAUC* = 0.03). Differences from the baseline were negligible for all other models.

Findings from feature engineering experiments used to assess the influence of encoding demographic and triplet information on model accuracy are also illustrated in Figs 4, 5 and 6. ML models encoding demographic information (i.e., *Labs+data* data subsets) showed higher accuracy than baseline ML models. Among ML models without filtered features, the performance of those encoding demographic information (i.e., *Labs+demo* data subsets) were 0.73 for logistic regression, 0.74 for gradient boosting, and 0.67 for neural network models. The magnitude of performance improvement of *Labs+demo* data subsets from the baseline were *ΔAUC* = 0.09 for logistic regression, *ΔAUC* = 0.05 for gradient boosting, and *ΔAUC* = 0.04 for neural network models. Among ML models with filtered features, the performance of models encoding demographic information were 0.72 for logistic regression, 0.73 for gradient boosting, and 0.67 for neural network models. The magnitude of the performance improvements from the baseline were *ΔAUC* = 0.08 for logistic regression, *ΔAUC* = 0.04 for gradient boosting, and *ΔAUC* = 0.04 for neural network models.

ML models encoding triplet information (i.e., *Labs+data+events* and *Labs+demo+events+triples* data subsets) showed higher accuracy than baseline ML models. Among ML models encoding triplet information, the top performing models were 0.73 for logistic regression, 0.75 for gradient boosting, and 0.68 for neural network models. For ML models without filtered features, when compared to baseline models, the performance improvements were *ΔAUC* = 0.7 for logistic regression, *ΔAUC* = 0.05 for gradient boosting, and *ΔAUC* = 0.02 for neural network models for *Labs+demo+events* data subsets. The same differences were observed for logistic regression and gradient boosting ML models with filtered features for *Labs+demo+events* data subsets. For the neural network model with filtered features for the *Labs+demo+events* data subset, *ΔAUC* = 0.05. For models with filtered features for *Labs+demo+events+triples* data subsets, when compared to baseline models, the performance improvements were *ΔAUC* = 0.8 for logistic regression, *ΔAUC* = 0.05 for gradient boosting, and *ΔAUC* = 0.02 for neural network models.

## Discussion

### Clinically meaningful triplets and utility of discriminative score

Eighty two clinically meaningful triplets were discovered for a severe asthma case study (S1 and S2 Tables). We found that the precision of *MI*_*score*_ rankings to discover those triplets was low to moderate for prescriptions (20% to 67%) and low for procedures (0% to 20%). This finding indicates that more work is needed in order to move from manual clinical review to an automated process. Through further assessment of *MI*_*score*_ rankings, we found that similar model performance for the mortality prediction task can be achieved with decreased model complexity (i.e., fewer features selected according to discriminative score, Figs 2, 3 and 4). These findings suggest that *MI*_*score*_ alone cannot replace clinical input, but that the use of *MI*_*score*_-informed rankings has potential to decrease model complexity at little cost to model performance.

### Model complexity and performance of machine learning models

We assessed *model complexity* for logistic regression and gradient boosting. We found decreases in model complexity with the use of fewer features informed by discriminative score and with clinical input (Table 4 and Table 5). We also found decreases in model complexity when demographic information was encoded. Results were mixed for models that encoded triplet information, with decreases in model complexity observed with *Labs+demo+events* data subsets, and an increase in complexity observed with one *Labs+demo+events+triples* data subset.

When considering model performance for approaches that decreased model complexity, we found that logistic regression, gradient boosting, and neural network ML models were robust to feature removal informed by discriminative score. We did however observe diminishing performance with fewer features. Among the machine learning models we explored, the neural network model was the most robust to feature removal.

For logistic regression, gradient boosting, neural network, and KNN models with filtered features, we found small differences in performance when compared to baseline ML models.(*ΔAUC* was ≤0.03 across all modeling approaches). For logistic regression, gradient boosting and neural network models with filtered features, we also found that encoding demographic and triplet information showed both decreases in model complexity and improvements in performance for the *Labs+demo* and *Labs+demo+events* subsets. The results were mixed for the *Labs+demo+events+triples* subsets, with observed decreases in model complexity and improvements in performance observed for the logistic regression and gradient boosting models with filtered features for the *Labs+demo+events+triples* subsets. For the neural network model with filtered features for the *Labs+demo+events+triples* subset, model complexity increased and the improvement in performance was very small.

### Limitations and implications for future work

Our use of the MIMIC-III dataset in this case study may influence generalizability of our approach to other clinical datasets given that the dataset derives exclusively from patients in intensive care settings. Because of this, information typically collected in routine outpatient settings, such as pulmonary function tests, would not be included in our model. This limitation was mitigated in part through our filtering approach that excluded routine features of intensive care.

There are also some limitations due to our approach to define triplets. First, when considering prescriptions as the anchor event for triplets, the half-life of the medications may be relevant. Our approach to discover triplets as lab-event-lab triples (i.e., laboratory results prior to and immediately following a prescription) may exclude some triplets relevant to medications with a longer half-life. Second, under circumstances where we used a default binning approach for laboratory results, extreme outliers skewed the boundaries. There are other approaches to discretize time series data such as SAX [24] and Temporal Discretization for Classification (TD4C) [25] methods that might be considered to overcome this limitation.

In addition, the performance of our ML approaches may be influenced by our cohort description. First, severe asthma patients were selected according to asthma medications and diagnoses listed for those patients. Our inclusion criteria may be improved by processing the free text “admission reason” to determine if asthma or asthma-like terms are mentioned. Second, we did not consider the presence of co-morbid conditions in our predictive modeling. Future work may draw from others to improve prediction or treatment guidelines for severe asthma. For example, there are many studies such as [26] that aim to detect factors that can predict asthma. We anticipate that such considerations can provide improved predictions over approaches explored here.

A major contribution of this work to the ML literature is our approach to incorporate clinical expertise. When reviewing ML and temporal data mining research broadly, we found that this was a gap. [4, 6, 8, 27–33]. Unlike with previous efforts, our experiments tested the impact of using a feature engineering approach to incorporate clinical input on model complexity and performance. We also provided a simplified framework to encode triplets (lab-event-lab patterns) for direct inclusion into our models that warrants further exploration. Furthermore, well-known feature selection methods such as Lasso [34] do not incorporate expert knowledge and rely on statistical methods to choose features. Findings from our work motivate future efforts to explore ways to use statistical methods together with approaches such ours that incorporate clinician-informed information on the relationship between clinical events and laboratory measurements into the machine learning process.

## Conclusion

This work explored a feature engineering approach with longitudinal data that enabled incorporating clinical input in ML models. We assessed the impact of two characteristics of the approach on the complexity and performance of ML models for a mortality prediction task for a severe asthma case study: ranking features by discriminative score (e.g., *MI*_*score*_ sum), and filtering laboratory features according to input on lab-event-lab triplets that are clinically meaningful. We found that ML models that use fewer input features selected based on discriminative score or according to which triplets are clinically meaningful, can both decrease model complexity with little cost to performance. Furthermore, for models with lower model complexity through the use of filtered features, the performance of ML models showed improvements from the baseline for data subsets that encode demographic and triplet information. Such approaches to reduce ML model complexity at little cost to performance warrant further comparison and consideration to combine with state-of-the-art feature selection methods, as well as exploration beyond the severe asthma case study.

## Supporting information

S1 Table Clinically meaningful prescription triplets ranked by discriminative score. (PDF)

S2 Table Clinically meaningful procedure triplets ranked by discriminative score. (PDF)

S3 Table Top 20 prescription triplets ranked by discriminative score. (PDF)

S4 Table Top 20 procedure triplets ranked by discriminative score. (PDF)

S5 Table Laboratory tests found in list of clinically meaningful triplets. (PDF)

S6 Table Forty-two laboratory tests used in gradient boosting experiments, sorted by weight. (PDF)

S7 Table Forty-two laboratory tests used in logistic regression experiments, sorted by weight. (PDF)
