# Supplementary material for: Feature engineering with clinical expert knowledge: A case study assessment of machine learning model complexity and performance
Source: PLoS One. 2020 Apr 23;15(4):e0231300. doi: 10.1371/journal.pone.0231300 (PMC7179831; doi:10.1371/journal.pone.0231300)
Supplement: S1 Table — (PDF) [file pone.0231300.s001.pdf]

S1 Table. Clinically meaningful prescription triplets ranked by discriminative score.

**Anchor prescription event**	**Laboratory test name**	MI_{score}
Xopenex	Oxygen saturation	0.170
Albuterol Sulfate	Bicarbonate	0.160
Salmeterol	pH	0.123
Xopenex	CO2 (ETCO2, PCO2, etc.)	0.079
Xopenex	Bicarbonate	0.064
Xopenex	Partial pressure of carbon dioxide	0.061
Salmeterol	CO2 (ETCO2, PCO2, etc.)	0.053
Methylprednisolone	Bicarbonate	0.038
Budesonide	Bicarbonate	0.033
Budesonide	Oxygen saturation	0.029
Xopenex	pH	0.023
Salmeterol	Bicarbonate	0.016
Dexamethasone	CO2 (ETCO2, PCO2, etc.)	0.015
Dexamethasone	Partial pressure of carbon dioxide	0.015
Dexamethasone	Positive end-expiratory pressure	0.012
Dexamethasone	Lactate	0.012
Albuterol	Oxygen saturation	0.008
Albuterol	Positive end-expiratory pressure	0.007
Salmeterol	Partial pressure of carbon dioxide	0.006
Dexamethasone	pH	0.006
Albuterol	CO2 (ETCO2, PCO2, etc.)	0.005
Albuterol	Bicarbonate	0.005
Albuterol	Partial pressure of carbon dioxide	0.004
Methylprednisolone	pH	0.003
Dexamethasone	Bicarbonate	0.002
Albuterol	pH	0.001
Albuterol Sulfate	pH	0

MI_{score} = mutual information score
