# Supplementary material for: Feature engineering with clinical expert knowledge: A case study assessment of machine learning model complexity and performance
Source: PLoS One. 2020 Apr 23;15(4):e0231300. doi: 10.1371/journal.pone.0231300 (PMC7179831; doi:10.1371/journal.pone.0231300)
Supplement: S2 Table — (PDF) [file pone.0231300.s002.pdf]

S2 Table. Clinically meaningful procedure triplets ranked by discriminative score.

**Anchor procedure event**	**Laboratory test name**	MI_{score}[1]
Unplanned Extubation (patient-initiated)	Partial pressure of carbon dioxide	0.15
Thoracentesis	CO2 (ETCO2, PCO2, etc.)	0.12
Unplanned Extubation (patient-initiated)	Bicarbonate	0.09
Percutaneous Tracheostomy	Bicarbonate	0.08
Thoracentesis	Partial pressure of carbon dioxide	0.08
Percutaneous Tracheostomy	Partial pressure of carbon dioxide	0.07
Unplanned Extubation (patient-initiated)	CO2 (ETCO2, PCO2, etc.)	0.06
Invasive Ventilation	Oxygen saturation	0.06
Intubation	Oxygen saturation	0.05
Percutaneous Tracheostomy	CO2 (ETCO2, PCO2, etc.)	0.05
Percutaneous Tracheostomy	pH	0.04
Thoracentesis	Bicarbonate	0.04
Thoracentesis	pH	0.03
Non-invasive Ventilation	Oxygen saturation	0.03
Extubation	Lactate	0.03
Intubation	Lactate	0.02
Cardioversion/Defibrillation	Partial pressure of carbon dioxide	0.02
Dialysis - CRRT	Blood urea nitrogen	0.02
Extubation	Oxygen saturation	0.02
Cardioversion/Defibrillation	Bicarbonate	0.02
Cardioversion/Defibrillation	pH	0.01
Non-invasive Ventilation	CO2 (ETCO2, PCO2, etc.)	0.01
Cardioversion/Defibrillation	CO2 (ETCO2, PCO2, etc.)	0.01
Dialysis Catheter	Potassium	0.01
Bronchoscopy	Partial pressure of carbon dioxide	0.01
Dialysis Catheter	Bicarbonate	0.01
Interventional Radiology	White blood cell count	0.01
Dialysis Catheter	pH	0.01
Cardioversion/Defibrillation	Lactate	0.01
Non-invasive Ventilation	Partial pressure of carbon dioxide	0.00
Non-invasive Ventilation	Bicarbonate	0.00
Extubation	pH	0.00
Extubation	Bicarbonate	0.00
Invasive Ventilation	Partial pressure of carbon dioxide	0.00
Dialysis - CRRT	pH	0.00
Dialysis - CRRT	Creatinine	0.00
Intubation	Bicarbonate	0.00
Invasive Ventilation	CO2 (ETCO2, PCO2, etc.)	0.00
Dialysis - CRRT	Bicarbonate	0.00
Intubation	CO2 (ETCO2, PCO2, etc.)	0.00
Invasive Ventilation	Lactate	0.00
Intubation	pH	0.00
Intubation	Partial pressure of carbon dioxide	0.00
Extubation	CO2 (ETCO2, PCO2, etc.)	0.00
Non-invasive Ventilation	pH	0.00
Bronchoscopy	pH	0.00
Non-invasive Ventilation	Lactate	0.00
Dialysis - CRRT	Potassium	0.00
Invasive Ventilation	Bicarbonate	0.00
Invasive Ventilation	pH	0.00
Bronchoscopy	CO2 (ETCO2, PCO2, etc.)	0.00
Extubation	Partial pressure of carbon dioxide	0.00
Dialysis Catheter	Creatinine	0.00
Dialysis Catheter	Blood urea nitrogen	0.00
Unplanned Extubation (patient-initiated)	pH	0.00

MI_{score} = mutual information score
