# Supplementary material for: Feature engineering with clinical expert knowledge: A case study assessment of machine learning model complexity and performance
Source: PLoS One. 2020 Apr 23;15(4):e0231300. doi: 10.1371/journal.pone.0231300 (PMC7179831; doi:10.1371/journal.pone.0231300)
Supplement: S3 Table — (PDF) [file pone.0231300.s003.pdf]

S3 Table. Top 20 prescription triplets ranked by discriminative score.

**Anchor prescription event**	**Laboratory test name**	MI_{score}	Relevant to case study?
Xopenex	Oxygen saturation	0.17	Y
Budesonide	Hemoglobin	0.16	N
Albuterol Sulfate	Bicarbonate	0.16	Y
Albuterol Sulfate	Sodium	0.15	N
Methylprednisolone	Glucose	0.14	N
Albuterol Sulfate	Glucose	0.14	N
Salmeterol	pH	0.12	Y
Budesonide	Mean corpuscular hemoglobin concentration	0.11	N
Budesonide	Bilirubin	0.10	N
Albuterol Sulfate	Blood urea nitrogen	0.09	N
Albuterol Sulfate	Mean corpuscular volume	0.09	N
Budesonide	Mean corpuscular hemoglobin	0.09	N
Xopenex	Monocytes	0.08	N
Methylprednisolone	Hematocrit	0.08	N
Albuterol Sulfate	Hematocrit	0.08	N
Albuterol Sulfate	Calcium	0.08	N
Budesonide	White blood cell count	0.08	N
Methylprednisolone	Magnesium	0.08	N
Methylprednisolone	Anion gap	0.08	N
Xopenex	CO2 (ETCO2, PCO2, etc.)	0.08	Y

MI_{score} = mutual information score
