# Supplementary material for: Feature engineering with clinical expert knowledge: A case study assessment of machine learning model complexity and performance
Source: PLoS One. 2020 Apr 23;15(4):e0231300. doi: 10.1371/journal.pone.0231300 (PMC7179831; doi:10.1371/journal.pone.0231300)
Supplement: S4 Table — (PDF) [file pone.0231300.s004.pdf]

S4 Table. Top 20 procedure triplets ranked by discriminative score.

**Anchor procedure**	**Laboratory test name event**	MI_{score}	Relevant to case study?
Extubation	Basophils	0.30	N
Peritoneal Dialysis	Bicarbonate	0.28	N
Peritoneal Dialysis	Prothrombin time	0.22	N
Nuclear Medicine	Mean corpuscular hemoglobin concentration	0.20	N
Unplanned Line/ Catheter Removal (Patient Initiated)	Glucose	0.18	N
Paracentesis	Glucose	0.17	N
Cardioversion/Defibrillation	Mean corpuscular hemoglobin concentration	0.16	N
Percutaneous Tracheostomy	Prothrombin time	0.16	N
Dialysis Catheter	Lactate dehydrogenase	0.15	N
Extubation	Positive end-expiratory pressure	0.15	N
Unplanned Extubation (patient-initiated)	Partial pressure of carbon dioxide	0.15	Y
Lumbar Puncture	Glucose	0.14	N
Unplanned Extubation (patient-initiated)	Mean corpuscular volume	0.14	N
Unplanned Extubation (patient-initiated)	Hemoglobin	0.13	N
Unplanned Extubation (patient-initiated)	Hematocrit	0.13	N
Lumbar Puncture	pH	0.13	N
Unplanned Extubation (patient-initiated)	Mean corpuscular hemoglobin concentration	0.12	N
Thoracentesis	CO2 (ETCO2, PCO2, etc.)	0.12	Y
Temporary Pacemaker Wires Discontinued	Hemoglobin	0.12	N
Unplanned Extubation (patient-initiated)	Glucose	0.11	N

MI_{score} = mutual information score
