# Supplementary material for: Feature engineering with clinical expert knowledge: A case study assessment of machine learning model complexity and performance
Source: PLoS One. 2020 Apr 23;15(4):e0231300. doi: 10.1371/journal.pone.0231300 (PMC7179831; doi:10.1371/journal.pone.0231300)
Supplement: S5 Table — (PDF) [file pone.0231300.s005.pdf]

S5 Table. Laboratory Tests Found in List of Clinically Meaningful triplets

Laboratory Test Name	Procedure triplets (N=55)	Prescription triplets (N=27)
Bicarbonate	10	7
Blood urea nitrogen	2	0
CO2 (ETCO2, PCO2, etc.)	9	4
Creatinine	2	0
Lactate	5	1
Oxygen saturation	4	3
Partial pressure of carbon dioxide	9	4
pH	11	6
Positive end-expiratory pressure	0	2
Potassium	2	0
White blood cell count	1	0
