# Supplementary material for: Feature engineering with clinical expert knowledge: A case study assessment of machine learning model complexity and performance
Source: PLoS One. 2020 Apr 23;15(4):e0231300. doi: 10.1371/journal.pone.0231300 (PMC7179831; doi:10.1371/journal.pone.0231300)
Supplement: S6 Table — (PDF) [file pone.0231300.s006.pdf]

S6 Table. Forty-two Laboratory Tests Used in Gradient Boosting Experiments, sorted by weight

Laboratory Test	Weight*	Relevant to case study?
Blood culture	0.08	N
Cholesterol	0.06	N
Red blood cell count	0.06	N
Creatinine	0.06	Y
Glucose	0.04	N
Lactate	0.04	Y
Calcium	0.04	N
Lactate dehydrogenase	0.03	N
Prothrombin time	0.03	N
pH	0.03	Y
Partial pressure of carbon dioxide	0.03	Y
Partial thromboplastin time	0.02	N
Potassium	0.02	Y
Eosinophils	0.02	N
Asparate aminotransferase	0.02	N
Blood urea nitrogen	0.02	Y
Hemoglobin	0.02	N
Sodium	0.02	N
Hematocrit	0.02	N
Mean corpuscular hemoglobin	0.02	N
Mean corpuscular hemoglobin concentration	0.02	N
Chloride	0.02	N
Neutrophils	0.02	N
Oxygen saturation	0.02	Y
Monocytes	0.02	N
Alkaline phosphate	0.02	N
Magnesium	0.02	N
Bilirubin	0.01	N
Alanine aminotransferase	0.01	N
Bicarbonate	0.01	Y
Lymphocytes	0.01	N
White blood cell count	0.01	Y
Albumin	0.01	N
Phosphate	0.01	N
Basophils	0.01	N
Mean corpuscular volume	0.01	N
Positive end-expiratory pressure	0.01	Y
Platelets	0.01	N
CO2 (ETCO2, PCO2, etc.)	0.01	Y
Anion gap	0.01	N
Troponin-I	0.01	N
Troponin-T	0.00	N

*Weight according to Gradient Boosting model
