# Supplementary material for: Feature engineering with clinical expert knowledge: A case study assessment of machine learning model complexity and performance
Source: PLoS One. 2020 Apr 23;15(4):e0231300. doi: 10.1371/journal.pone.0231300 (PMC7179831; doi:10.1371/journal.pone.0231300)
Supplement: S7 Table — (PDF) [file pone.0231300.s007.pdf]

S7 Table. Forty-two Laboratory Tests Used in Logistic Regression Experiments, sorted by weight

Laboratory Test	Weight*	Relevant to case study?
Mean corpuscular hemoglobin concentration	3.89	N
Bicarbonate	3.35	Y
Hematocrit	3.21	N
Anion gap	3.08	N
Glucose	3.05	N
Prothrombin time	2.69	N
Chloride	2.65	N
Calcium	2.59	N
Mean corpuscular volume	2.56	N
Hemoglobin	2.49	N
Asparate aminotransferase	2.49	N
Sodium	2.33	N
White blood cell count	2.32	Y
Mean corpuscular hemoglobin	2.30	N
Lactate dehydrogenase	2.03	N
Phosphate	2.01	N
CO2 (ETCO2* PCO2* etc.)	1.98	Y
Lymphocytes	1.93	N
Monocytes	1.85	N
Potassium	1.78	Y
Basophils	1.61	N
Partial pressure of carbon dioxide	1.59	Y
Blood urea nitrogen	1.50	Y
Oxygen saturation	1.49	Y
Magnesium	1.40	N
Red blood cell count	1.32	N
Neutrophils	1.30	N
pH	1.24	Y
Platelets	1.23	N
Positive end-expiratory pressure	1.19	Y
Bilirubin	1.18	N
Alanine aminotransferase	1.03	N
Alkaline phosphate	0.93	N
Lactate	0.87	Y
Creatinine	0.87	Y
Partial thromboplastin time	0.77	N
Cholesterol	0.69	N
Eosinophils	0.14	N
Troponin-I	0.09	N
Troponin-T	0.06	N
Albumin	0.05	N
Blood culture	0.00	N

*Weight is MI_{score} sum
